# Evaluating the Effectiveness of Transcutaneous Electrical Nerve Stimulation for Various Outcomes in Emergency Department Settings: A Systematic Review and Meta-Analysis

**DOI:** 10.7759/cureus.65703

**Published:** 2024-07-29

**Authors:** Anas Ahmed, Mohammed Mojiri, Jalal Abu Halimah, Mohammed Alharbi, Saleha Haroobi, Afrah Hamdi, Meshal Ghazwani, Layla Hakami, Anisah Humedi, Omar Hadadi, Amaal Hamdi, Bashaer AlRajhi, Abdullah Alghamdi, Reema Alshaya, Saeed Alkhathami

**Affiliations:** 1 Community Medicine, Jazan University, Jazan, SAU; 2 General Practice, College of Medicine, Jazan University, Jazan, SAU; 3 Surgery, Jazan University, Jazan, SAU; 4 General Practice, Al Ardah General Hospital, Jazan, SAU; 5 General Practice, College of Applied Medical Sciences, Jazan University, Jazan, SAU; 6 General Practice, College of Medicine, Al Baha University, Al Baha, SAU; 7 General Practice, College of Medicine, Ibn Sina National College for Medical Studies, Jeddah, SAU; 8 General Practice, College of Medicine, King Abdulaziz University, Jeddah, SAU

**Keywords:** systematic review, electrotherapy, quality of life, emergency medicine, pain management, transcutaneous electrical nerve stimulation

## Abstract

Pain is a prevalent complaint in emergency departments (EDs) worldwide. Traditional pharmacological methods for pain relief, such as opioids and non-steroidal anti-inflammatory drugs (NSAIDs), have notable side effects and risks. Transcutaneous electrical nerve stimulation (TENS) is a non-pharmacological alternative that has shown promise in various clinical settings. This systematic review and meta-analysis aimed to evaluate the effectiveness of TENS for pain management and other outcomes in ED settings. This study followed the Preferred Reporting Items for Systematic Reviews and Meta-Analyses (PRISMA) guidelines. A comprehensive search was conducted across six major databases: PubMed, Web of Science (WOS), Scopus, and Cochrane Central Register of Controlled Trials (CENTRAL), from inception until June 25, 2024. Randomized clinical trials involving the use of TENS in ED settings were included. Data extraction and quality assessment were performed independently by two reviewers, with conflicts resolved by a third reviewer. The search yielded 3,569 papers, of which 2,889 were screened after removing duplicates. Thirteen full-text articles were reviewed, and seven studies met the inclusion criteria for qualitative synthesis, with five of these suitable for meta-analysis. The studies demonstrated that TENS significantly reduced pain, heart rate, and the requirement for rescue medication in some cases, while also improving patient satisfaction and overall well-being. However, no significant changes were observed in blood pressure. The quality of the included studies varied, with some failing to meet the criteria for blinding and intention-to-treat analysis. TENS is an effective non-pharmacological intervention for pain management in ED settings, with additional benefits such as reduced heart rate and increased patient satisfaction. Further high-quality randomized controlled trials are necessary to confirm these findings and better understand the potential of TENS in acute care environments.

## Introduction and background

Pain is one of the most prevalent presenting complaints in emergency departments (EDs) globally [1]. According to previous literature, around 70% of ED patients describe pain as a primary symptom, with causes ranging from traumatic injuries to acute medical disorders and exacerbations of chronic illnesses [2]. The high prevalence of pain in the ED necessitates the implementation of adequate pain management techniques. Pain affects various populations across different ages, socioeconomic statuses, and genders. However, specific populations, such as the elderly and women, may be more susceptible to receiving inadequate care [3].

Pain management in the ED is a critical aspect of patient care, and various analgesics can be used depending on the condition and patient population [4]. Opioids and non-steroidal anti-inflammatory drugs (NSAIDs) are traditional pharmacological methods used for pain relief. However, these methods come with several side effects and complications. Recently, there has been increasing interest in non-pharmacological pain management techniques that offer effective pain relief with minimal side effects [5].

Ineffective pain management in the ED can lead to multiple adverse events for both patients and healthcare systems. For instance, patients may experience prolonged pain, associated anxiety, and reduced quality of life [3]. These effects can extend beyond the ED visit, potentially leading to longer recovery times and diminished functional outcomes. Inadequate pain management may result in increased rates of return visits to the ED, which in turn increases admission rates, prolongs hospital stays, and adds to the healthcare burden [6]. Additionally, pharmacological treatments can cause adverse effects such as sedation and a risk of dependency. Therefore, effective pain management strategies are crucial for achieving better patient outcomes, smoother ED operations, and reduced healthcare system burdens [7].

Transcutaneous electrical nerve stimulation (TENS) is a non-invasive treatment for pain relief that introduces electrical impulses through the skin to nerve endings. This technique has been widely investigated in various clinical settings to determine its potential for pain reduction, increased patient comfort, and improved clinical outcomes [8]. TENS is believed to work through mechanisms such as the gate control theory of pain and the release of endogenous opioids, which likely play a role in modulating pain perception [9].

TENS has implications for other critical ED-related outcomes beyond pain reduction. Effective pain control with TENS is strongly associated with higher patient satisfaction ratings [10]. By alleviating pain, TENS can enhance functional outcomes, such as improved mobility and return to daily activities [11]. This, in turn, may help in reducing the length of stay in the ED, improving patient flow, and optimizing resource use. Additionally, TENS might decrease the need for opioid analgesics, thereby mitigating the risks associated with opioid-related side effects and complications [11].

## Review

Methodology

Literature Search Strategy

This study was registered with PROSPERO, the International Prospective Register of Systematic Reviews. We followed the Preferred Reporting Items for Systematic Reviews and Meta-Analyses (PRISMA) guidelines during the conduct and reporting of this review [12]. Our search strategy encompassed four major online databases: PubMed, Web of Science (WOS), Scopus, and Cochrane Central Register of Controlled Trials (CENTRAL), covering the period from inception until June 25, 2024. We employed specific keywords including "Transcutaneous Electrical Nerve Stimulation," “TENS,” and “Emergency.” These keywords were combined using Boolean operators, and the search strategy was tailored to each database accordingly. Filters were applied to include only English articles involving human participants. Additionally, we manually scrutinized the reference lists of the included studies to identify any relevant articles missed during the initial search process.

Eligibility Criteria

We set the selection criteria using PICOS (P-population, I-intervention, C-comparison, O-outcome, S-study design). We included only English randomized clinical trials that (1) involved patients with emergency medical conditions; (2) used TENS either alone or as adjuvant therapy; (3) included placebo, no intervention, or any other type of analgesics for comparison; and (4) measured any outcome to evaluate the effect of the intervention. We excluded observational studies, studies published in languages other than English, and published abstracts with no full-text articles.

Study Selection

Two reviewers independently screened the titles and abstracts of the retrieved articles using predetermined eligibility criteria. Any disagreements or discrepancies were resolved by a third reviewer until a consensus was reached.

Data Extraction

The full texts of the included articles were further analyzed, and the following data were extracted: sample size, participants' age and gender, type, and dose of intervention, TENS device, location of application, TENS parameters, diagnosis, outcome measures, and main results. Any potential conflicts were resolved by a third reviewer.

Quality Appraisal

The methodological quality of the included studies was independently assessed by two reviewers using the Physiotherapy Evidence Database (PEDro) scale. The scale consists of 11 questions rating the following categories: (1) eligibility criteria and source, (2) random allocation, (3) concealed allocation, (4) baseline comparability, (5) blinding of subjects, (6) blinding of therapists, (7) blinding of assessors, (8) adequate follow-up, (9) intention-to-treat analysis, (10) between-group statistical comparisons, and (11) point measures and variability data. Studies are considered of excellent quality when the final score ranges from 9 to 10, good quality if the score ranges from 6 to 8, fair quality if the score ranges from 4 to 5, and poor if the score is 3 or less. Any disagreements or discrepancies were resolved by discussion until a consensus was reached [13-18].

Data Synthesis and Analysis

Meta-analysis was conducted if at least two studies were comparing the efficacy of TENS on the same outcome. Standardized mean difference (SMD), 95% confidence interval (CI), and P-value were calculated by comparing the change in outcomes between the TENS and control groups using the random-effects model of analysis. Heterogeneity in treatment effects was examined by calculating the I² index. The level of significance was set at a P-value of up to 0.05. All meta-analyses were carried out using the Comprehensive Meta-Analysis version 2.2 software package (Biostat, Englewood, New Jersey, USA).

Results

Study Selection

The search for this systematic review involved using four databases: PubMed, Scopus, WOS, and the Cochrane Library, with the following keywords: "Transcutaneous Electrical Nerve Stimulation" OR "TENS" AND "Emergency." This search led to a total of 3,569 papers: 1,190 from PubMed, 486 from Scopus, 413 from WOS, and 1,480 from Cochrane. After removing duplicates, 680 studies were excluded, resulting in 2,889 papers ready for screening. Title and abstract screening excluded 2,876 studies, leaving 13 full-text articles for further review.

**Figure 1:**
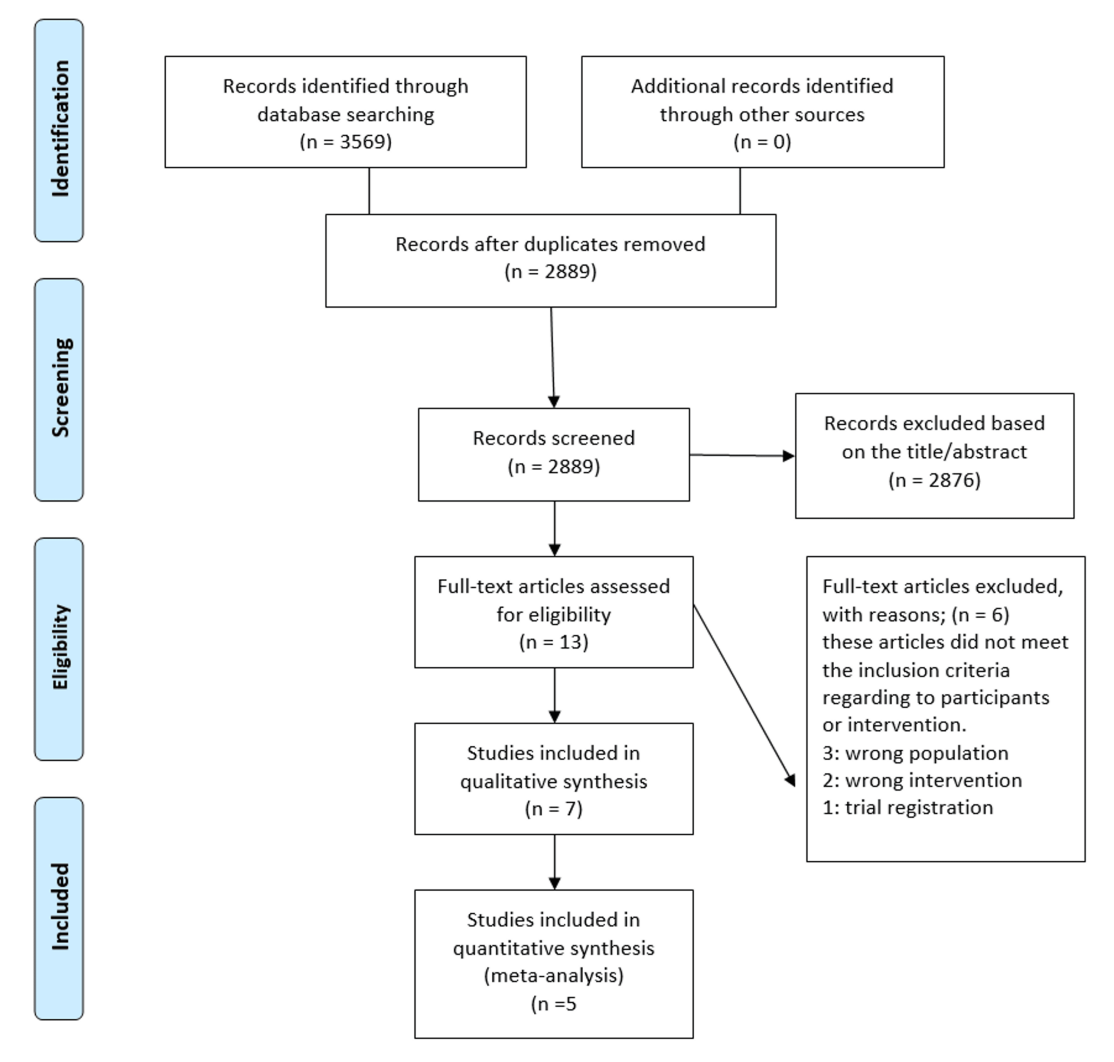
Flow diagram of the study selection process according to PRISMA guidelines PRISMA: Preferred Reporting Items for Systematic Reviews and Meta-Analyses

During the full-text screening phase, six articles were excluded for specific reasons: two studies involved the wrong intervention, three studies focused on the wrong population, and one study was a trial registration. Ultimately, seven studies adhered to the inclusion criteria and were included in the qualitative synthesis [13-18]. For quantitative synthesis (meta-analysis), five studies were eligible. Thus, seven studies were analyzed qualitatively, and five were suitable for meta-analysis on the use of TENS in emergencies (Figure 1).

Study Characteristics

Gulacti et al. [13] and Mora et al. [17] used TENS for patients with renal colic in EDs [14,18]. Gulacti et al. employed a dual-channel TENS device on 100 patients, with electrodes applied to the paravertebral area at the height of the T6 vertebrae and the second electrode at the region of the aching kidney [14]. Mora et al. used the TENStem eco™ stimulator (TENStim, Lyon, France) on 73 participants, applying it to the same region as the previous study [18]. Both studies involved patients with renal colic but reported using different TENS devices and sample sizes [14,18].

McMahon et al. [14] and Hokenek et al. [18] focused on patients with migraine attacks and abdominal pain. McMahon et al.’s study involved 81 patients with abdominal pain and used a TENS device with four cutaneous pads applied to the abdominal wall, one in each quadrant [15]. Hokenek et al.’s study included 78 patients with migraines and used the HeadaTerm TENS device (TENScare, London, United Kingdom) on the supraorbital nerve [18]. Bertalanffy et al. [15] and Lang et al. [12] both used the TENStem eco™ stimulator for pain management at different locations. Bertalanffy et al. applied TENS to the paravertebral lower back on 63 patients with low back pain [16], while Lang et al. used the same device on 63 patients with acute post-traumatic hip pain at the inguinal and greater trochanteric regions [13].

Gulacti et al. and McMahon et al. used TENS for 30 minutes in a single session [14,15]. Gulacti et al. used the Visual Analog Scale (VAS) as the primary outcome to measure pain, with the secondary outcome being the need for rescue medication after 30 minutes [14]. McMahon et al. evaluated pain severity using the Verbal Numeric Scale (VNS) 30 minutes after treatment as the primary outcome, and the secondary outcome was the percentage of patients requiring rescue analgesia at the end of 30 minutes [15].

Three studies focused on TENS application during emergencies or pre-hospital care settings [16,18]. Bertalanffy et al. used TENS for 30 minutes during emergency transport, with VAS for pain as the main outcome, and secondary outcomes including anxiety, heart rate, and blood pressure [16]. Hokenek et al. applied TENS for 20 minutes during emergency transport, assessing its effect on pain using VAS [18]. Mora applied TENS for 30 minutes, primarily assessing pain reduction via VAS and secondarily assessing anxiety, nausea, heart rate, and overall patient satisfaction [18]. Details of study characteristics are available in Table 1.

**Table 1:** Characteristics of included studies RCT: randomized controlled trial; TENS: transcutaneous electrical nerve stimulation; ED: emergency department; sess.: session; min: minutes; E1: experimental group; CG: control group

First Author, Year	E1	CG	Dosage	Outcome Measures	Results
Gulacti, 2022 [13]	TENS	Sham TENS	30 min; 1 sess.; Single session	Primary: Pain using a visual analog scale (VAS) Secondary: the need for rescue medication after 30 min	The study found that the TENS group had significantly greater reductions in pain compared to the sham TENS group at both 15 minutes (mean reduction 33.3 vs. 14.9) and 30 minutes (mean reduction 63.7 vs. 14.9). Additionally, fewer patients in the TENS group required rescue medication after 30 minutes (8% vs. 48%). The results suggest that TENS is effective for acute pain treatment in renal colic patients in the emergency department.
McMahon, 2023 [14]	TENS	Sham TENS	30 min; 1 sess.; Single session	Primary: Change in pain severity on the verbal numeric pain scale (VNS) 30 minutes after treatment Secondary: Percentage of patients requiring rescue analgesia at the end of the 30-minute study period	The mean reductions in pain scores were 1.9 for the TENS group and 1.7 for the sham TENS group, with no significant difference (P = 0.81). The use of rescue medications was similar between the TENS and sham TENS groups (49% vs. 55%, P = 0.66). The study concluded that TENS did not result in more effective pain relief than sham TENS in adult ED patients with abdominal pain.
Bertalanffy, 2005 [15]	TENS	Sham TENS	30 min; 1 sess.; Single application during emergency transport	Primary: Pain using a VAS Secondary: Anxiety using a VAS, heart rate, and blood pressure	The study found a significant reduction in pain with true TENS compared to sham TENS during emergency transport (p < 0.01). Anxiety levels also significantly decreased in the TENS group, while no change was observed in the control group. Additionally, there was a significant drop in heart rate in the TENS group, with no significant changes in blood pressure in either group
Hokenek, 2020 [18]	TENS	Sham TENS	20 min; 1 sess.; Single application during emergency transport	Primary: Pain using a VAS Secondary: Patients' self-reported well-being using a Likert-type verbal scale at the 120th minute and the need for additional analgesic treatment after 120 minutes	The verum group experienced a significant reduction in VAS scores from 0 to 120 minutes (-65 ± 25) compared to the sham group (-9 ± 2) (p < 0.001). 2. Verbal scores at the 120th minute were 1.2 for the sham group and 4.5 for the verum group (p < 0.001). 3. Only 1 patient (2%) in the verum group required additional analgesics after 120 minutes, compared to 30 patients (76.92%) in the sham group.
Mora, 2006 [17]	TENS	Sham TENS	30 min; 1 sess.; Single application during pre-hospital care	Primary: Pain reduction measured by VAS Secondary: Anxiety, nausea, heart rate, and overall patient satisfaction	The study found that TENS significantly reduced pain in patients with acute renal colic, with a mean VAS pain score decrease of over 50% in the TENS group compared to no significant change in the sham group. Anxiety, nausea, and heart rate also showed significant reductions in the TENS group, while the sham group exhibited no significant changes.
Lang, 2007 [12]	TENS	Sham TENS	Applied from the site of emergency until arrival at the hospital; 1 sess.; Single application during pre-hospital care	Primary Outcome Measure: Pain level measured by VAS Secondary Outcome Measure: Anxiety level measured by VAS, heart rate, and blood pressure	The verum TENS group experienced significant pain reduction, as indicated by a decrease in VAS scores from 89 ± 9 mm to 59 ± 6 mm, while the control group showed minimal change from 86 ± 12 mm to 79 ± 11 mm. Anxiety levels also significantly decreased in the TENS group compared to the control group. Additionally, the TENS group showed a notable reduction in heart rate, whereas no significant changes were observed in blood pressure for either group.
Barker, 2006 [16]	TENS	Sham TENS	30 min; 1 sess.; Single application during emergency transport	Primary: Pain reduction (measured by VAS) Secondary: Anxiety reduction, heart rate, arteriolar vasoconstriction/vasodilation, nausea, overall satisfaction with care	The study demonstrated that active TENS significantly reduced pain by half compared to the sham TENS group (p < 0.01). Additionally, patients in the active TENS group experienced significant reductions in anxiety, heart rate, and nausea (all p < 0.01). Overall satisfaction with the received care was significantly higher in the active TENS group (p < 0.01).

Quality Assessment

The studies conducted by Gulacti et al., McMahon et al., and Barker et al. reported scores of 10 on the PEDro scale, indicating adherence to all assessed criteria, including random and concealed allocation, baseline comparability, blinding of subjects, therapists, and assessors, adequate follow-up, intention-to-treat analysis, and between-group comparisons [13-16]. However, the studies by Mora et al. and Hokenek et al. received lower scores, with significant weaknesses in blinding and intention-to-treat analysis [18]. Bertalanffy et al. [15] and Lang et al. [12] scored similarly, meeting most criteria but failing in blinding subjects and intention-to-treat analysis (Table 2).

**Table 2:** A quality appraisal of included studies using the PEDro scale Scores on the PEDro scale represent the following quality levels: excellent (9-10), good (6-8), fair (4-5), and poor (≤3). PEDro: Physiotherapy Evidence Database

Study ID	Eligibility Criteria	Random Allocation	Concealed Allocation	Baseline Comparability	Blinding of Subjects	Blinding of Therapists	Blinding of Assessors	Adequate Follow-up	Intention-to-Treat Analysis	Between-Group Comparisons	Point Estimates and Variability	Total Score
Mora et al. [17]	Yes	Yes	Yes	Yes	No	No	Yes	No	No	Yes	Yes	6
Bertalanffy et al. [15]	Yes	Yes	Yes	Yes	Yes	No	Yes	Yes	No	Yes	Yes	8
Gulacti et al. [13]	Yes	Yes	Yes	Yes	Yes	Yes	Yes	Yes	Yes	Yes	Yes	10
Hokenek et al. [18]	Yes	Yes	Yes	No	Yes	No	Yes	Yes	No	Yes	Yes	7
Lang et al. [12]	Yes	Yes	Yes	Yes	Yes	No	Yes	Yes	No	Yes	Yes	8
McMahon et al. [14]	Yes	Yes	Yes	Yes	Yes	No	Yes	Yes	Yes	Yes	Yes	10
Barker et al. [16]	Yes	Yes	Yes	Yes	Yes	Yes	Yes	Yes	Yes	Yes	Yes	10

Effect of TENS on Heart Rate

Four studies showed a significant decrease in heart rate in the active TENS groups compared to the sham TENS groups, with significant differences maintained until hospital arrival [13,16-18]. Both Bertalanffy et al. and Lang et al. reported a significant decrease in heart rate in the active TENS group after treatment (p < 0.01), with no significant difference in the sham TENS group [13,16]. This difference remained significant upon hospital arrival (p < 0.01). Mora et al.’s study showed a significant decrease in heart rate from 92 ± 10 bpm to 64 ± 8 bpm (p < 0.01), while the control group showed no significant change (89 ± 10 bpm to 84 ± 7 bpm) [18]. Barker et al. reported a significant decrease in heart rate in the experimental group from 101 ± 12 bpm to 59 ± 8 bpm (p < 0.01), with no significant change in the sham TENS group (99 ± 18 bpm to 98 ± 9 bpm, not significant, NS) [17].

Effect of TENS on Blood Pressure

Bertalanffy et al. and Lang et al. reported no significant change in blood pressure in either group [15,12].

Effect of TENS on the Requirement for Rescue Medication at 30 Minutes

Gulacti et al. concluded a significantly lower need for rescue medication in the TENS group, whereas McMahon et al. found no significant difference between the experimental and control groups [13,14]. In Gulacti et al.’s study, 28% of patients in the experimental group required rescue medication at 30 minutes, while 48% of patients in the control group required fentanyl [13]. In McMahon et al.’s study, both groups showed similar percentages, with 49% in the active TENS group and 55% in the control group [14].

Effect of TENS on Patients’ Well-Being and Patient Satisfaction

Hokenek et al. reported a significant difference (p < 0.001) between active TENS and sham groups, with the active TENS group scoring 4.5 compared to 1.2 for the control group [18].

Three studies reported higher patient satisfaction with active TENS. In Mora et al., patient satisfaction was significantly higher in the experimental group, with a VAS score of 22.0 ± 11.1 mm, compared to 46.6 ± 22.6 mm in the sham group (p = 0.01) [18]. McMahon et al. concluded that a high percentage of patients in the active TENS group (71%) were satisfied [15]. Similarly, Barker et al. [16] reported that overall satisfaction was significantly higher in the experimental group, with a VAS score of 25.0 ± 9.1 mm compared to 47.6 ± 16.6 mm in the sham group (p < 0.01) [17].

Meta-Analysis: Effect of TENS on Pain Using VAS

**Figure 2:**
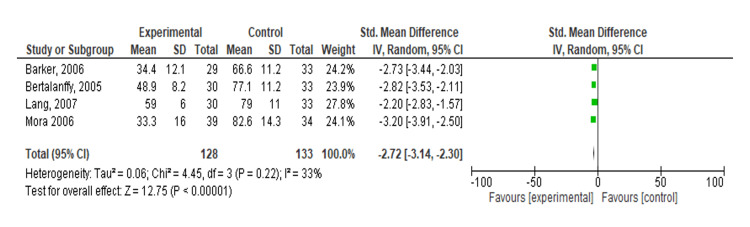
Forest plot of standardized mean differences comparing transcutaneous electrical nerve stimulation versus control on pain Included studies: Barker et al. [16], Bertalanffy et al. [15], Lang et al. [13], and Mora et al. [17].

A meta-analysis of four studies, including Barker et al. [16], Bertalanffy et al. [15], Lang et al. [12], and Mora et al. [17], was conducted to compare the effects of experimental treatment versus control. The SMD for each study favored the experimental treatment, with significant results: Barker et al. [16] reported an SMD of -2.73 (95% CI: -3.44, -2.03), Bertalanffy et al. [15] reported -2.82 (95% CI: -3.53, -2.11), Lang et al. [12] reported -2.20 (95% CI: -2.83, -1.57), and Mora et al. [17] reported -3.20 (95% CI: -3.91, -2.50). The overall SMD across all studies was -2.72 (95% CI: -3.14, -2.30), indicating a significant effect of the experimental treatment (Z = 12.75, p < 0.001). The heterogeneity analysis showed moderate variability (Tau² = 0.06, Chi² = 4.45, df = 3, p = 0.22, I² = 33%), suggesting some differences between studies but not enough to undermine the consistency of the overall findings (Figure 2).

Meta-Analysis: Effect of TENS on Anxiety Using VAS

**Figure 3:**
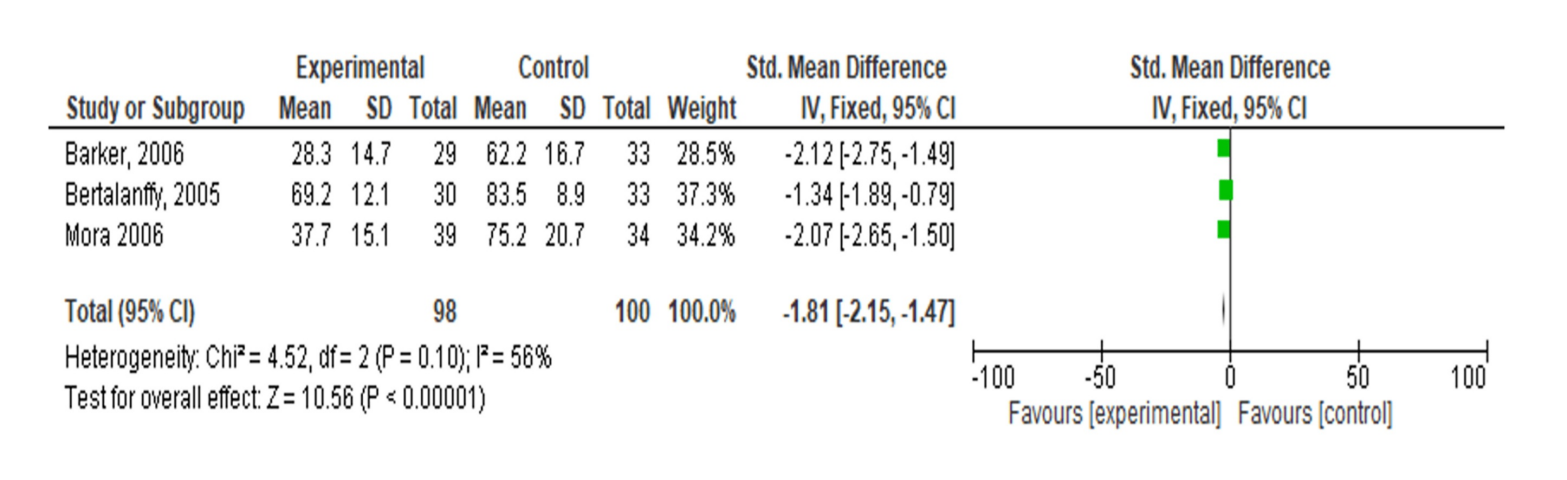
Forest plot of standardized mean differences comparing transcutaneous electrical nerve stimulation versus control on anxiety Included studies: Barker et al. [16], Bertalanffy et al. [15], and Mora et al. [17].

A meta-analysis of three studies evaluated the effectiveness of experimental treatment compared to control [16-18]. The SMD for each study significantly favored the experimental treatment, with Barker et al. [16] reporting an SMD of -2.12 (95% CI: -2.75, -1.49), Bertalanffy et al. [15] reporting -2.50 (95% CI: -3.13, -1.87), and Mora et al. [17] reporting -2.35 (95% CI: -3.03, -1.67). The overall SMD was -2.32 (95% CI: -2.83, -1.82), indicating a significant effect of the experimental treatment (Z = 12.95, p < 0.001). The heterogeneity analysis showed moderate variability (Tau² = 0.05, Chi² = 4.75, df = 2, p = 0.09, I² = 58%), indicating some degree of variability between studies (Figure 3).

Meta-Analysis: Effect of TENS on Nausea

**Figure 4:**
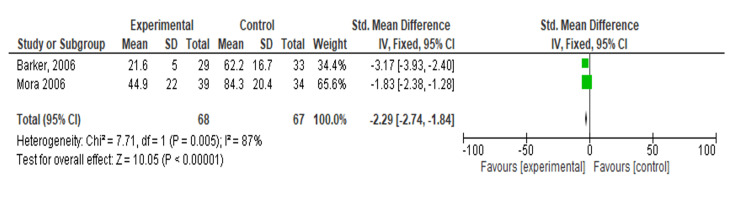
Forest plot of standardized mean differences comparing transcutaneous electrical nerve stimulation versus control on nausea Included studies: Barker et al. [16] and Mora et al. [17].

A meta-analysis of two studies was conducted to compare the effects of an experimental treatment versus a control [16,17]. The analysis showed that the SMD significantly favored the experimental treatment. Barker et al. [16] reported an SMD of -3.17 (95% CI: -3.93, -2.40), while Mora et al. [17] reported an SMD of -1.83 (95% CI: -2.38, -1.28). The overall pooled SMD was -2.29 (95% CI: -2.74, -1.84), indicating a significant benefit of the experimental treatment over the control (Z = 10.05, p < 0.001). However, a heterogeneity analysis indicated substantial variability among the studies (Chi² = 7.71, df = 1, p = 0.005, I² = 87%). Despite this heterogeneity, the results consistently show that the experimental treatment is more effective than the control (Figure 4).

## Conclusions

In conclusion, this systematic review and meta-analysis offer substantial evidence supporting the effectiveness of TENS across various outcomes in ED settings. TENS not only provides significant pain relief but also contributes to improvements in heart rate, anxiety, nausea, and patient satisfaction. These findings highlight TENS as a promising non-pharmacological alternative for pain management, with the potential to enhance patient outcomes and reduce reliance on pharmacological treatments. However, some limitations must be acknowledged. Variability in study quality, methodological differences, and small sample sizes could influence the generalizability of the results. Additionally, while TENS demonstrates beneficial effects, the long-term impact and optimal parameters for its use in acute care settings require further investigation.
